# Machine learning‐based radiomics nomograms to predict number of fields in postoperative IMRT for breast cancer

**DOI:** 10.1002/acm2.14194

**Published:** 2023-11-01

**Authors:** Yichen Mao, Wenyi Di, Dan Zong, Zhongde Mu, Xia He

**Affiliations:** ^1^ The Affiliated Cancer Hospital of Nanjing Medical University & Jiangsu Institute of Cancer Research & Jiangsu Cancer Hospital Nanjing China

**Keywords:** breast cancer, machine learning, number of fields, radiomics, radiotherapy

## Abstract

**Background:**

Breast cancer is now the most commonly diagnosed cancer in women worldwide. Radiotherapy is an important part of the treatment for breast cancer, while setting proper number of fields dramatically affects the benefits one can receive. Machine learning and radiomics have been widely investigated in the management of breast cancer. This study aims to provide models to predict the best number of fields based on machine learning and improve the prediction performance by adding clinical factors.

**Methods:**

Two‐hundred forty‐two breast cancer patients were retrospectively enrolled for this study, all of whom received postoperative intensity modulated radiation therapy. The patients were randomized into a training set and a validation set at a ratio of 7:3. Radiomics shape features were extracted for eight machine learning algorithms to predict the number of fields. Univariate and multivariable logistic regression were implemented to screen clinical factors. A combined model of rad‐score and clinical factors were finally constructed. The area under receiver operating characteristic curve, precision, recall, F1 measure and accuracy were used to evaluate the model.

**Results:**

Random Forest outperformed from eight machine learning algorithms while predicting the number of fields. Prediction performance of the radiomics model was better than the clinical model, while the predictive nomogram combining the rad‐score and clinical factors performed the best.

**Conclusions:**

The model combining rad‐score and clinical factors performed the best. Nomograms constructed from the combined models can be of reliable references for medical dosimetrists.

## 1 INTRODUCTION

Breast cancer constitutes the highest prevalence of cancer diagnosis among the global female population, accounting for 25.8% of the total incidence of new malignancies.^1^ Surgery is the main treatment modality for breast cancer,^2^, ^3^ and among which breast‐conserving surgery with postoperative adjuvant radiotherapy has become the standard treatment for early‐stage breast cancer.^4^, ^5^ Furthermore, early‐stage breast cancer patients achieved the same therapeutic effect by breast‐conserving surgery combined with radiotherapy as the radical mastectomy.^6^ It is reported that breast‐conserving surgery combined with radiotherapy could reduce the 10‐year recurrence rate from 35% to 19%, and reduce the 10‐year breast cancer mortality rate from 17% to 14%.^7^ Therefore, postoperative adjuvant radiotherapy plays an important role in the comprehensive treatment of breast cancer.

The number of fields plays a crucial role in designing IMRT plans especially for breast cancer patients,^8^ because it influences the target conformity and dose uniformity which are essential for achieving optimal treatment outcomes.^9^, ^10^ However, determining the optimal number of radiation fields is challenging due to the big variations of breast shape and lymph status among patients. In addition, it is usually a time‐consuming work for medical dosimetrists to achieve an adequate plan because trial‐and‐error is an inevitable process during plan design for breast cancer, while other plans may only need an even‐distributed 9‐fields plan such as head and neck tumors.^11^ Although other techniques such as Volumetric‐modulated Arc Therapy (VMAT) could bypass the process of setting number of fields, it is more common that Linacs (medical linear accelerators) have the function of intensity‐modulated radiotherapy (IMRT) rather than VMAT due to the fact that IMRT is usually a default setting on Linacs instead of VMAT. Furthermore, for some hospitals in underdeveloped areas of the world, there are no VMAT function in their Linacs but only IMRT. Therefore, determining the optimal number of radiation fields would be the first priority work for breast cancer IMRT planning.

Recently, artificial intelligence (AI) has become a powerful tool for designing breast cancer radiation treatment plans. It is reported that automatic planning of breast cancer radiotherapy is faster than manual planning and can achieve comparable dose distribution.^12^, ^13^ Setting the number of fields is the first step and an inevitable part to design an automatic planning system. However, it is still a challenge for most automatic planning systems to generate the optimal number of fields.^14^

Radiomics is a technique that could extract large numbers of features from medical images using high‐throughput computing and transform them into high‐dimensional data which can be investigated as clinical decision supports.^15^, ^16^ Recently, radiomics has been widely used in the diagnosis, staging and prognosis of breast cancer.^17^, ^18^ Besides, it can also quantify the morphological characteristics of the tumor, which are key factors for determining the number of radiation fields during the planning design process.^8^, ^19^ Therefore, radiomics‐based analysis may have the potential to predict the optimal number of fields.

To sum up, it can be assumed that radiomics could act as an important part in breast cancer plan design along with AI algorithms. Therefore, in this research we explored the feasibility of predicting the optimal number of fields for postoperative adjuvant radiotherapy of breast cancer patients using radiomics features. By the aid of AI, such as machine learning tools, nomograms were developed based on radiomics scores and clinical features. It is expected that this research could be a helpful reference for medical dosimetrists in breast cancer plan design.

## 2 MATERIAL AND METHODS

### 2.1 Patients

**FIGURE 1:**
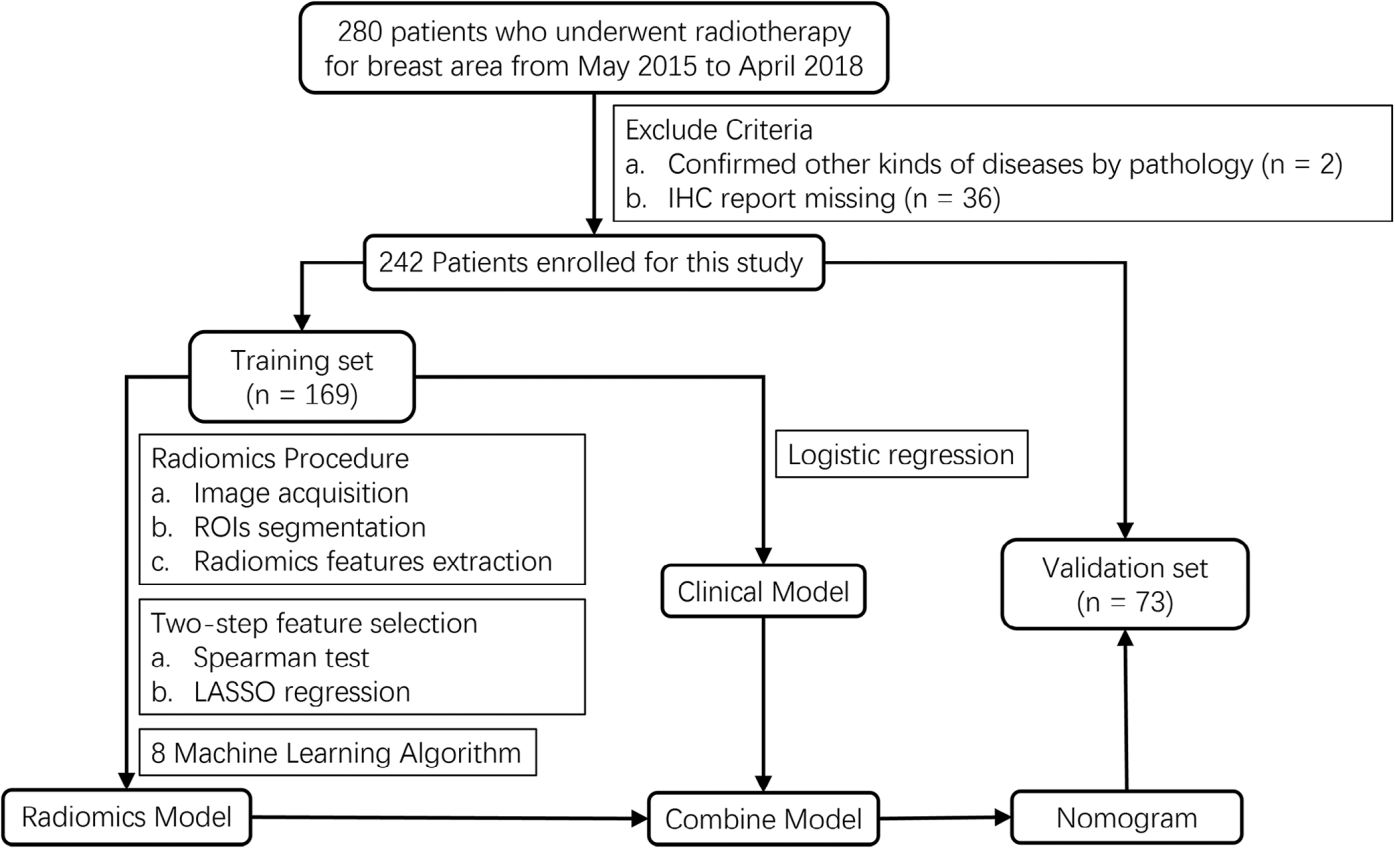
Flow chart of patient recruitment and model establishment.

This study was approved by the review committee in our institution. Due to the retrospective nature of the study, the requirements for informed consent were waived. Two‐hundred eighty patients with postoperative breast cancer were retrospectively reviewed, who underwent IMRT in our institution between May 2015 and April 2018. Patients who met any of these criteria were excluded: (a) lack of pathological confirmation of breast cancer, (b) absence of immunohistochemistry (IHC) records (Figure 1). Finally, a total of 242 patients were enrolled for this study. They were then randomly divided into a training set and a validation set in a ratio of 7:3 (169:73).

The clinical variables were collected from patients’ clinical records, including age, gender, TNM stage, histological subtype, surgery type and IHC. TNM stages were evaluated base on the eighth edition of the American Joint Committee on Cancer (AJCC) staging system. Histological subtypes were divided as invasive ductal carcinoma (IDC) and others. IHC including the status of ER, PR, HER2 and Ki67. CT images and radiotherapy plans were collected from Eclipse (V15.6, Varian Medical Systems, USA). The CT scanning matrix was set to 512 × 512 pixels, and the slice thickness was 5 mm and the tube voltage was 120 kV.

### 2.2 ROIs definition and feature extraction

**FIGURE 2:**
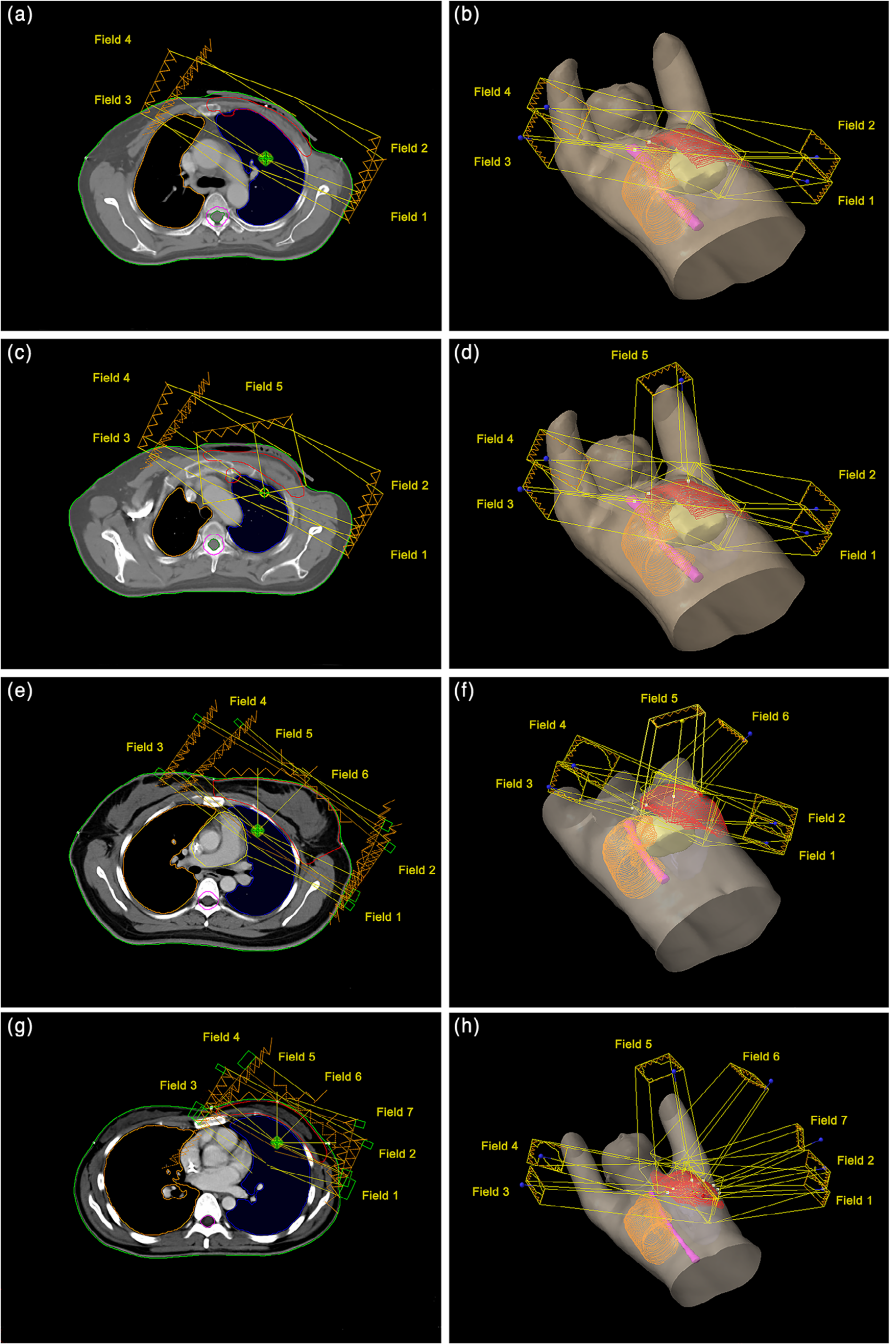
Plans of different number of fields. (a) 2D view of a 4‐fields plan. (b) 3D view of a 4‐fields plan. (c) 2D view of a 5‐fields plan. (d) 3D view of a 5‐fields plan. (e) 2D view of a 6‐fields plan. (f) 3D view of a 6‐fields plan. (g) 2D view of a 7‐fields plan. (h) 3D view of a 7‐fields plan.

The gross target volume (GTV) and organ‐at‐risk (OAR) were manually delineated by medical doctors with more than 7 years working experience in the department of radiotherapy. The accuracy of each segmentation was reviewed by senior medical doctors. Organs delineated by medical doctors are treated as region of interests (ROIs), and options for the number of fields are shown in Figure 2. Both of ROIs definition and radiotherapy planning were performed on the Eclipse V15.6 treatment plan system.

Radiomics features were extracted from ROIs by the Pyradiomics package, an open‐source package of Python. This package could extract signal strength features, texture features, shape features, and higher‐order features from the ROIs. Defined features were in compliance with feature definitions described by Image Biomarker Standardization Initiative (IBSI).^20^ Only shape features of Radiomics were collected because all the other features were closely related to image characteristics rather than planning needs. Several kinds of Hausdorff Distances among ROIs were also calculated and collected as features by Plastimatch, which is an open‐source software for volumetric image processing^21^ (Table S1).

### 2.3 Feature selection

The plans for radiotherapy were made by medical dosimetrists according to as‐given prescriptions. The number of fields were determined by the shape of GTV segmentations in order to meet the prescription requirements. Both of shape features and Hausdorff Distances were taken into account for the purpose of predicting number of fields. A two‐step feature selection method was adopted. Firstly, The Spearman test was conducted among features, and the one with a lower correlation to the number of fields would be removed from the pair if it had a correlation coefficient greater than 0.8. Secondly, the least absolute shrinkage and selection operator (LASSO) algorithm was implemented which could automatically tune the number of features by a recursive feature elimination. The regularization parameter λ was adjusted by 10‐fold cross validation. At last, features with non‐zero coefficients were reserved.^22^

### 2.4 Model establishment

Before modeling, Synthetic Minority Over‐sampling Technique (SMOTE) was adopted for data equilibrium.^23^ A total of eight popular machine learning (ML) algorithms‐decision tree (DT), random forest (RF), support vector machine (SVM), naïve bayes (NB), K‐nearest neighbors (KNN), logistic regression (LR), extreme gradient boosting (XGBoost) and adaptive boosting (AdaBoost)‐were investigated on the training set using the one‐versus‐all multiclass classification method. Leave‐one‐out cross‐validation (LOOCV) was used to construct machine learning models. For each model, precision, recall, F1 measure and accuracy were calculated to assess the prediction performance. Finally, the rad‐score were generated using the Logit function for further analysis.

Chi‐square test and Mann–Whitney *U*‐test or Student's *t*‐test were performed for categorical variables and continuous variables, respectively. Univariate and multivariable logistic regression were implemented to exclude the features without significance and to determine the final clinical characteristics that were used to establish the clinical model.

A combined model of the rad‐score from the radiomics model and clinical signatures from the clinical model was constructed by the LR algorithm to provide nomograms for predicting number of fields. Calibration curves were used to assess the agreement between predictions and observations in different percentiles of the predicted values. Clinical impact curve (CIC) was used to evaluate the clinical benefits of the nomogram.^24^

### 2.5 Statistical analysis

All statistical analysis was conducted using R software (version 4.2.2; http://www.Rproject.org). Categorical variables were compared by the chi‐square test. The Shapiro–Wilk's test was performed to assess the normality of continuous variables. Mann–Whitney U‐test was used to test the feature significance of variables with the null hypothesis rejection. Univariate and multivariate analyses were carried out for clinical model establishment. A two‐sided *p*‐value < 0.05 was considered statistically significant.

## 3 RESULTS

### 3.1 Patients’ characteristics

A total of 242 breast cancer patients who underwent postoperative IMRT were retrospectively enrolled in this study, with an average age of 49.2 ± 9.06. There were 56 4‐fields plans, 47 5‐fields plans, 68 6‐fields plans and 71 7‐fields plans. The detailed clinical characteristics of the training set and the validation set are shown in Table 1. Patients were separated with no significant differences (*p* > 0.05).

**TABLE 1:** Detailed characteristics of all patients.

Characteristics	Training set	Validation set	*p*‐value
Age (mean, SD)	49.3 (8.95)	49.0 (9.39)	0.89
T (*n*, %)			0.77
1	80 (47.3%)	37 (50.7%)	
2	80 (47.3)	34 (46.6%)	
3	7 (4%)	1 (1.4%)	
4	2 (1%)	1 (1.4%)	
N (*n*, %)			0.33
0	82 (48.5%)	29 (39.7%)	
1	52 (30.8%)	28 (38.4%)	
2	24 (14.2%)	8 (11.0%)	
3	11 (6.5%)	8 (11.0%)	
M (*n*, %)			1
0	165 (97.6%)	72 (98.6%)	
1	4 (2.4%)	1 (1.4%)	
ER (*n*, %)			0.55
Positive	116 (68.6%)	47 (64.4%)	
Negative	53 (31.4%)	26 (35.6%)	
PR (*n*, %)			0.20
Positive	106 (62.7%)	39 (53.4%)	
Negative	63 (37.3%)	34 (46.6%)	
HER2 (*n*, %)			0.40
Positive	87 (51.5%)	42 (57.5%)	
Negative	82 (48.5%)	31 (42.5%)	
Ki67 (*n*, %)			0.77
<30%	108 (63.9%)	45 (61.6%)	
≥30%	61 (36.1%)	28 (38.4%)	
Subtype (*n*, %)			0.38
IDC	148 (87.6%)	67 (91.8%)	
Others	21 (12.4%)	6 (8.2%)	
Affected side (*n*, %)			0.78
L	90 (53.3%)	41 (56.2%)	
R	79 (46.7%)	32 (43.8%)	
Surgery (*n*, %)			0.49
BC	90 (53.3%)	35 (47.9%)	
RM	79 (46.7%)	38 (52.1%)	
Number of fields (*n*, %)			0.38
4	44 (26.0%)	12 (16.4%)	
5	33 (19.5%)	14 (19.2%)	
6	46 (27.2%)	22 (30.1%)	
7	46 (27.2%)	25 (34.2%)	

Abbreviation: SD, Standard Deviation.

### 3.2 Radiomics feature selection

**FIGURE 3:**
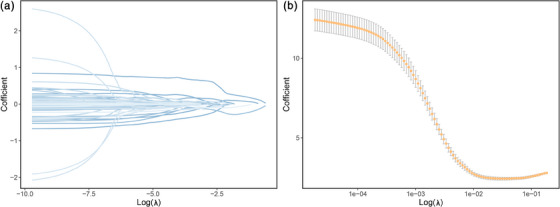
Radiomics features selection by LASSO. (a) Radiomics feature coefficient reduction path curves. Six non‐zero factors were selected at last. (b) The coefficient lambda of the penalty term in LASSO was seen as a hyperparameter and was tuned via the 10‐fold cross validation. LASSO, least absolute shrinkage and selection operator.

**FIGURE 4:**
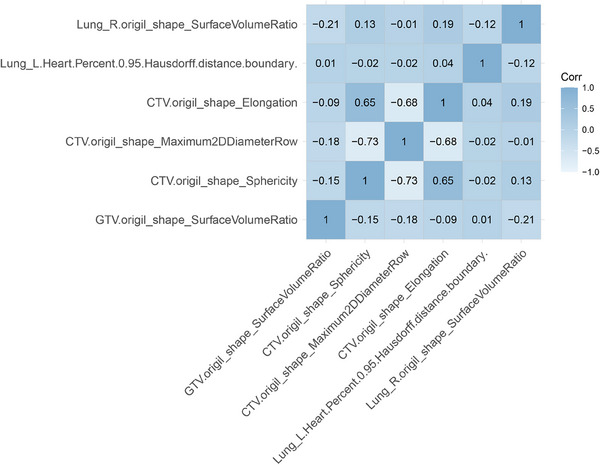
Correlation among the six selected features.

A total of 267 radiomics features were extracted from the ROIs in CT images. There were 73 features left after Spearman test, and 6 were selected finally after LASSO (Figure 3 and Table S2). The correlation plot of the six selected features is shown in Figure 4.

### 3.3 Machine learning model construction

**FIGURE 5:**
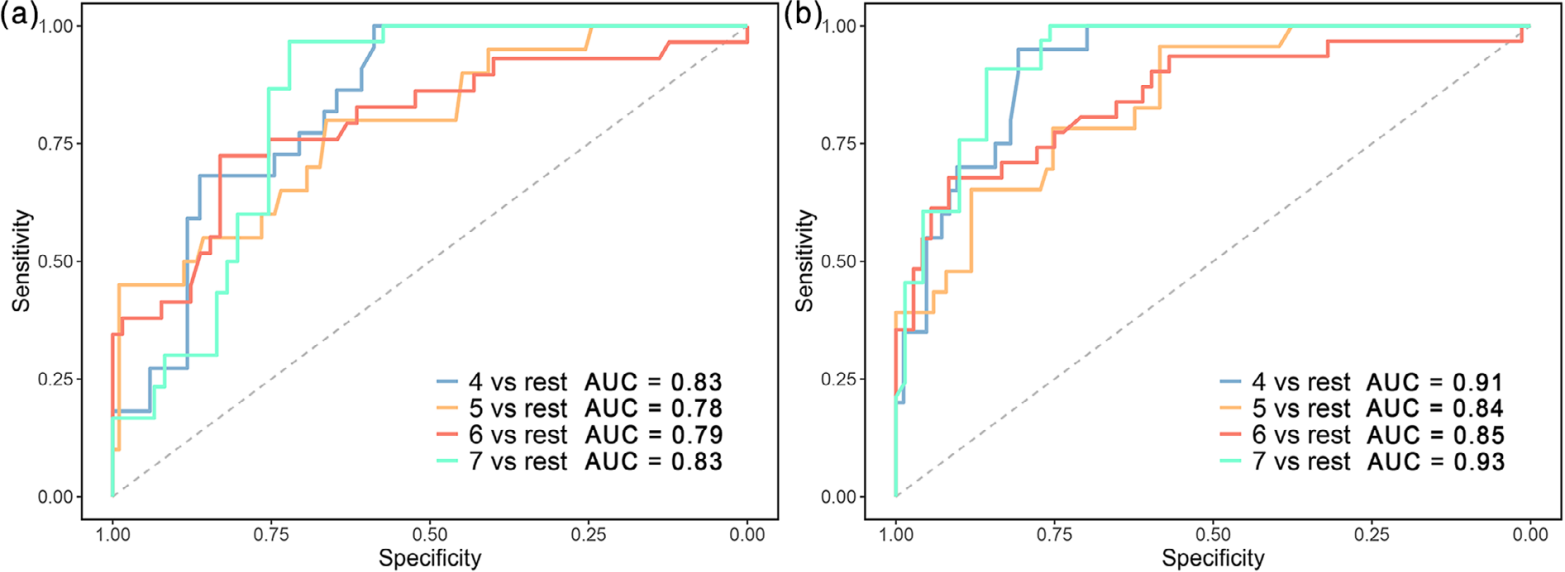
(a) ROC curves of RF in different number of fields plan prediction. (b) ROC curves of the combined model in different number of fields plan prediction. RF, random forest; ROC, receiver operating characteristic curve.

For each machine learning algorithm, macro precision, recall, F1 measure and accuracy were calculated (Table 2). The RF model had the best overall performance than the other seven algorithms and performed well on each classifier (Figure 5a and Table S3). Therefore, RF model was considered as the optimal radiomics model to generate the rad‐score. The distribution of rad‐score showed that the different classifications of number of fields have independent rad‐score (Figure S1).

**TABLE 2:** Overall performances of the eight machine learning classifiers.

Classifier	Precision	Recall	F1	Accuracy
**RF**	0.74	0.73	0.72	0.76
**KNN**	0.50	0.51	0.49	0.52
**NB**	0.46	0.50	0.47	0.52
**XGB**	0.70	0.70	0.69	0.70
**AB**	0.73	0.72	0.72	0.75
**SVM**	0.32	0.40	0.36	0.44
**LR**	0.41	0.46	0.42	0.46
**DT**	0.40	0.50	0.43	0.52

Abbreviations: DT, algorithms‐decision tree; KNN, K‐nearest neighbors; LR, logistic regression; NB, naïve bayes; RF, random forest; SVM, support vector machine; XGB, extreme gradient boosting.

### 3.4 Clinical model construction

The results of the univariate and multivariable LR are listed in Table 3 and Table S4. T stage, N stage and surgery type (*p* < 0.05) were determined as the variables for clinical model.

**TABLE 3:** Clinical risk factors for 4‐fields plan.

	Univariate			Multivariable		
Characteristics	OR	95%CI	*p*‐Value	OR	95%CI	*p‐*Value
Age	0.96	0.93–0.99	0.02	0.99	0.94–1.03	0.6
T (1, others)	0.29	0.16–0.52	<0.01	0.47	0.23–1.12	0.04
N (0, others)	0.06	0.03–0.13	<0.01	0.14	0.05–0.38	<0.01
ER (Positive, Negative)	2.54	1.33–4.85	<0.01	2.53	0.58–11.10	0.2
PR (Positive, Negative)	2.42	1.34–4.38	<0.01	0.93	0.24–3.61	>0.9
HER2 (Positive, Negative)	0.54	0.31–0.94	0.03	0.77	0.37–1.61	0.5
Ki67 (< 30%, ≥30%)	0.45	0.26–0.78	<0.01	0.59	0.28–1.25	0.2
Subtype (IDC, others)	2.22	1.02–4.84	0.05	/	/	/
Surgery (BC, RM)	0.01	0.00–0.06	<0.01	0.01	0.00–0.11	<0.01

Abbreviations: CI, confidence interval; OR, odd ratio.

### 3.5 Combined model establishment

**FIGURE 6:**
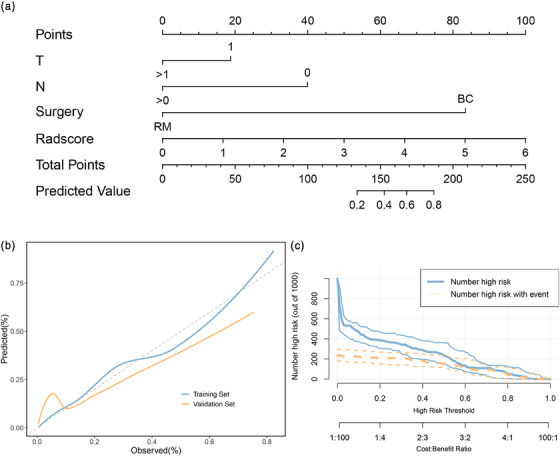
(a) An individualized nomogram based on rad‐score and clinical features for 4‐fields plan. (b) Calibration curve of the nomogram. (c) Clinical impact curves of the nomogram.

The radiomics model showed better performance than the clinical model. Rad‐score were then added to the clinical model to establish a combined model. The combined model showed good performance (AUC = 0.91, AUC = 0.84, AUC = 0.85, AUC = 0.93) on the validation set (Figure 5b). Nomograms were then built based on the combined model (Figure 6a). The overall performance of combined model was higher than that of the other two models (Table 4). The calibration curves showed that the nomogram was close to ideal on the validation set (Figure 6b). The CIC curve showed that the nomogram provides good clinical benefits (Figure 6c). The results of other models can be found in Figure S2, Figure S3 and Figure S4.

**TABLE 4:** Overall performances of the radiomics, clinical and combined model.

Classifier	Precision	Recall	F1	Accuracy
**Radiomics**	0.74	0.73	0.72	0.76
**Clinical**	0.52	0.5	0.45	0.53
**Combined**	0.79	0.79	0.78	0.80

## 4 DISCUSSION

In this study we investigated the potential of using radiomics features to predict the optimal number of fields for postoperative adjuvant breast cancer radiotherapy. Besides, we also explored clinical factors that were related to the number of fields. The results showed that the model based on clinical features and rad‐score could offer a reliable reference for determining the adequate number of fields.

For clinical model, it was shown that N stage plays a key role in distinguishing 4‐fields, 5‐fields, and 7‐fields plans. Patients with N0 stage are likely to be given 4‐fields plans. However, patients with lymph node metastasis are inclined to be treated with 5‐fields or 7‐fields plans. The possible reason is that, due to the lymph node area included in the GTV, it is not enough for 4‐fields plans yield the same dose homogeneity as that of 5‐fields or 7‐fields plans. In brief, N stage determines whether the lymph node should be included in the GTV or not, and large or small GTV eventually gives rise to how many fields should be used in the radiotherapy plans.

T stage and surgery type are also related to the optimal number of fields. Specifically, patients who underwent breast‐conserving surgery are more likely to be given 4‐fields plans, while those who underwent radical mastectomy are more suitable for 6‐fields and 7‐fields plans. This may be due to the target area after radical surgery is more irregular than breast‐conversing surgery. Furthermore, the dose conformality within the target area is a challenge with a 4‐fields plan. Previous reports also showed that the conformality within the target area can be improved by adding more fields.^25^ All these clinical factors, which may be related to number of fields, still need further exploration to reveal the interrelationship.

In recent years, radiomics has been widely used in diagnosis, staging, and prognosis prediction of breast cancer.^18^, ^26^ In addition, radiomics models can predict the risk of lymph node metastasis in breast cancer, thereby guiding the segmentation of radiotherapy target volumes.^27^ By contrast, it is still unclear whether radiomics is helpful or not in radiotherapy treatment planning. In this study, we designed prediction models based on radiomics shape features and Hausdorff Distances to predict the optimal number of fields. Shape features quantitatively described the shape of ROIs, and Hausdorff Distances are used to measure the relative positions between two ROIs. This is also the basis for dosimetrists to determine how many fields should be used at the very beginning. All these data should be carefully treated by choosing appropriate methods. Compared with clinical model, radiomics model works better at predicting the number of fields (Table 4).

In order to further improve the performance, a combined model based on rad‐score and clinical factors was established. As we had expected, the predictive ability of the combined model was better than both the radiomics model and clinical model. When predicting 5‐fields plans, AUC of the combined model was 0.84, while the radiomics model was 0.78. For ease of clinical use, the combined model was presented in nomograms. By checking these nomograms, dosimetrists could quickly determine the number of fields in radiotherapy treatment planning. Therefore, these nomograms could reduce repetitive work, and provide valuable references especially for dosimetrists’ beginners. In the meanwhile, the model itself can improve the function of setting the number of fields automatically for auto‐planning systems.

Nevertheless, there are still some limitations of this study. Firstly, our research was based on a small and retrospective dataset from a single center, which may limit its generalizability and reliability. Prospective, multicenter and large‐scale studies are needed to validate our findings. Secondly, GTVs were contoured by different experts who may have different opinions and criteria. Thirdly, only shape features of radiomics were collected as number of fields predictors which may hinder the performance of this powerful tool. It is expected that more comprehensive and robust features could be involved in future research. Fourthly, although we included the TNM stage to represent the target area, this may not always directly correspond to the drawn targets as there could be some elective coverage, which could potentially affect the accuracy of the model. And it is not only the number of fields but also gantry angles that are crucial factors for an adequate breast cancer radiotherapy plan. Such parameters should also be involved in the future to enrich our study and improve model accuracy.

## 5 CONCLUSION

In conclusion, combined with rad‐score and clinical factors, clinical‐radiomics nomograms were generated to estimate the optimal number of fields for post‐operative breast cancer radiotherapy. The predicting performance of the nomogram is satisfying. It is hoped that the nomograms would be a powerful tool in facilitating plan design and therefore reduce medical dosimetrists’ workloads. It is also believed that the combined models in this research would have potential usage in automatic radiotherapy planning.

## AUTHOR CONTRIBUTIONS

Zhongde Mu contributed substantially to the conception and design of the work. Yichen Mao and Wenyi Di participated in the acquisition, analysis and interpretation of data for the work. Zhongde Mu and Yichen Mao drafted the work and revised it critically for important intellectual content. All authors gave final approval of the version to be submitted for review and agreed to be accountable for all aspects of the work in ensuring that questions related to the accuracy or integrity of any part of the work are appropriately investigated and resolved.

## CONFLICT OF INTEREST STATEMENT

The authors have no conflicts of interest to declare.

## Supporting information

Supporting Information

Supporting Information
