# Supplementary material for: Machine learning‐based radiomics nomograms to predict number of fields in postoperative IMRT for breast cancer
Source: J Appl Clin Med Phys. 2023 Nov 1;25(3):e14194. doi: 10.1002/acm2.14194 (PMC10930011; doi:10.1002/acm2.14194)
Supplement: Supplementary file 1 — Supporting Information [file ACM2-25-e14194-s002.docx]

**Table S1** Hausdorff Distance definitions.

| **Type** | **Definition** | **Targets** |
| --- | --- | --- |
| bidirectional | the maximum of the one-sided Hausdorff distances in both directions | GTV, CTV, Left Lung, Right Lung, Heart, Spinal Cord, Healthy Breast. |
| average | the average of the one-sided Hausdorff distances in both directions |  |
| max average | the maximum of the average distances from each point in one set to the other set |  |
| fractional | the maximum distance from 95% of points in one set to the closest point in the other set |  |
| boundary | the bidirectional Hausdorff distance between the boundaries of the sets, instead of the whole sets. |  |
| average boundary | the average Hausdorff distance between the boundaries of the sets. |  |
| max average boundary | the max average Hausdorff distance between the boundaries of the sets. |  |
| fractional boundary | the fractional Hausdorff distance between the boundaries of the sets. |  |

**Table S2** Radiomics features selected for model establishment.

| **Feature** | **Coefficient** |
| --- | --- |
| GTV.origil_shape_SurfaceVolumeRatio | -0.09096919 |
| CTV.origil_shape_Sphericity | -0.10390647 |
| CTV.origil_shape_Maximum2DDiameterRow | 0.37046074 |
| CTV.origil_shape_Elongation | -0.18657074 |
| Lung_R.origil_shape_SurfaceVolumeRatio | 0.10160226 |
| Lung_L.Heart.Percent.0.95.Hausdorff.distance.boundary. | 0.12366044 |

**Table S3** Performances of RF in each classification on validation set.

| **Classifier** | **AUC** | **Accuracy** | **Sensitivity** | **Specificity** |
| --- | --- | --- | --- | --- |
| **4-vs-rest** | 0.83 | 0.87 | 0.95 | 0.79 |
| **5-vs-rest** | 0.78 | 0.88 | 0.90 | 0.45 |
| **6-vs-rest** | 0.79 | 0.78 | 0.89 | 0.61 |
| **7-vs-rest** | 0.83 | 0.90 | 0.93 | 0.86 |

**Table S4** Clinical risk factors for **(A)**5-field, **(B)**6-field and **(C)**7-field plan.

**(A)**

| **Characteristics** | **Univariate** | | | **Multivariable** | | |
| --- | --- | --- | --- | --- | --- | --- |
|  | **OR** | **95%CI** | **p** | **OR** | **95%CI** | **p** |
| Age | 1.01 | 0.99,1.05 | 0.3 |  |  |  |
| T (1, others) | 0.68 | 0.59,1.56 | <0.01 | 0.99 | 0.05,1.79 | 0.03 |
| N (0, others) | 3.53 | 2.09,6.07 | <0.01 | 2.87 | 1.55,5.38 | <0.01 |
| ER (Positive, Negative) | 1.11 | 0.64,1.92 | 0.7 | / | / | / |
| PR (Positive, Negative) | 1.02 | 0.61,1.72 | >0.9 | / | / | / |
| HER2 (Positive, Negative) | 1.59 | 0.95,2.67 | 0.08 | / | / | / |
| Ki67 (<30%, ≥30%) | 0.69 | 0.41,1.17 | 0.2 | / | / | / |
| Subtype (IDC, others) | 1.51 | 0.63,3.80 | 0.4 | / | / | / |
| Surgery (BC, RM) | 2.75 | 1.62,4.72 | <0.01 | 1.1 | 0.53,2.27 | 0.8 |

**(B)**

| **Characteristics** | **Univariate** | | | **Multivariable** | | |
| --- | --- | --- | --- | --- | --- | --- |
|  | **OR** | **95%CI** | **p** | **OR** | **95%CI** | **p** |
| Age | 0.99 | 0.96,1.02 | 0.5 |  |  |  |
| T (1, others) | 2.36 | 0.21,4.61 | <0.01 | 1.63 | 0.32,2.24 | 0.02 |
| N (0, others) | 0.41 | 0.24,0.69 | <0.01 | 0.79 | 0.41,1.55 | 0.5 |
| ER (Positive, Negative) | 0.85 | 0.50,1.45 | 0.6 |  |  |  |
| PR (Positive, Negative) | 1.09 | 0.65,1.83 | 0.7 |  |  |  |
| HER2 (Positive, Negative) | 0.52 | 0.31,0.87 | 0.05 |  |  |  |
| Ki67 (<30%, ≥30%) | 1.27 | 0.75,2.17 | 0.4 |  |  |  |
| Subtype (IDC, others) | 0.17 | 0.04,0.52 | <0.01 | 0.36 | 0.20,0.64 | 0.2 |
| Surgery (BC, RM) | 3.32 | 1.19,6.55 | <0.01 | 1.37 | 0.17,2.80 | 0.01 |

**(C)**

| **Characteristics** | **Univariate** | | | **Multivariable** | | |
| --- | --- | --- | --- | --- | --- | --- |
|  | **OR** | **95%CI** | **p** | **OR** | **95%CI** | **p** |
| Age | 0.97 | 0.95,1.00 | 0.08 |  |  |  |
| T (1, others) | 2.34 | 1.20,4.58 | <0.01 | 1.86 | 0.42,2.81 | 0.7 |
| N (0, others) | 2.32 | 1.06,5.17 | <0.01 | 1.13 | 0.56,2.25 | <0.01 |
| ER (Positive, Negative) | 0.7 | 0.41,1.18 | 0.2 |  |  |  |
| PR (Positive, Negative) | 0.72 | 0.43,1.19 | 0.2 |  |  |  |
| HER2 (Positive, Negative) | 1.87 | 1.10,3.20 | 0.02 | 1.51 | 0.80,2.85 | 0.2 |
| Ki67 (<30%, ≥30%) | 1.44 | 0.85,2.45 | 0.2 |  |  |  |
| Subtype (IDC, others) | 0.59 | 0.25,1.34 | 0.2 |  |  |  |
| Surgery (BC, RM) | 2.18 | 0.10,4.31 | <0.01 | 1.45 | 0.21,1.98 | 0.04 |
