# Supplementary material for: Machine learning‐based radiomics nomograms to predict number of fields in postoperative IMRT for breast cancer
Source: J Appl Clin Med Phys. 2023 Nov 1;25(3):e14194. doi: 10.1002/acm2.14194 (PMC10930011; doi:10.1002/acm2.14194)
Supplement: Supplementary file 2 — Supporting Information [file ACM2-25-e14194-s001.docx]

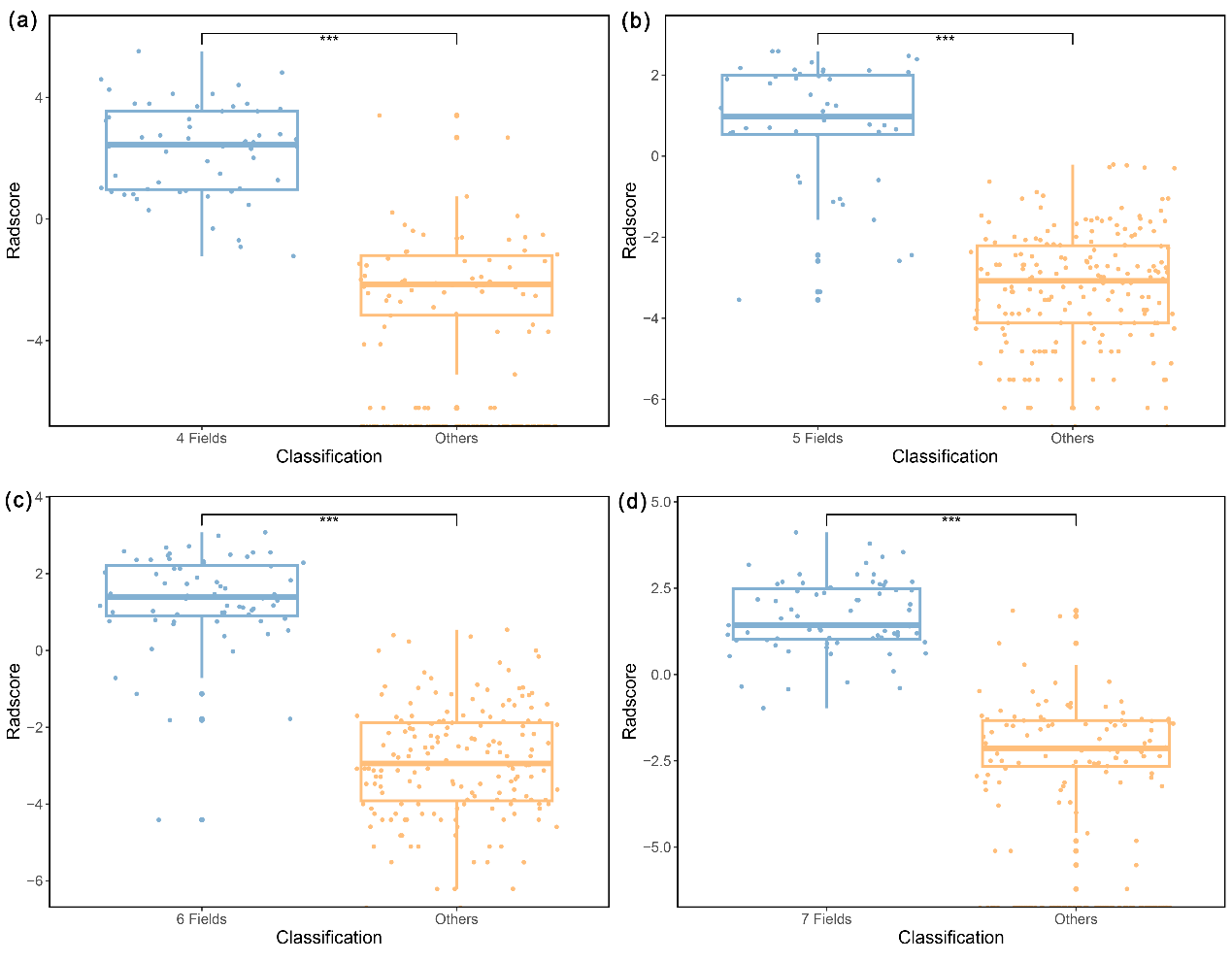


**Figure S1** The distribution of the rad-score and the Mann–Whitney U-test results in each classification. ***: p <0.01.


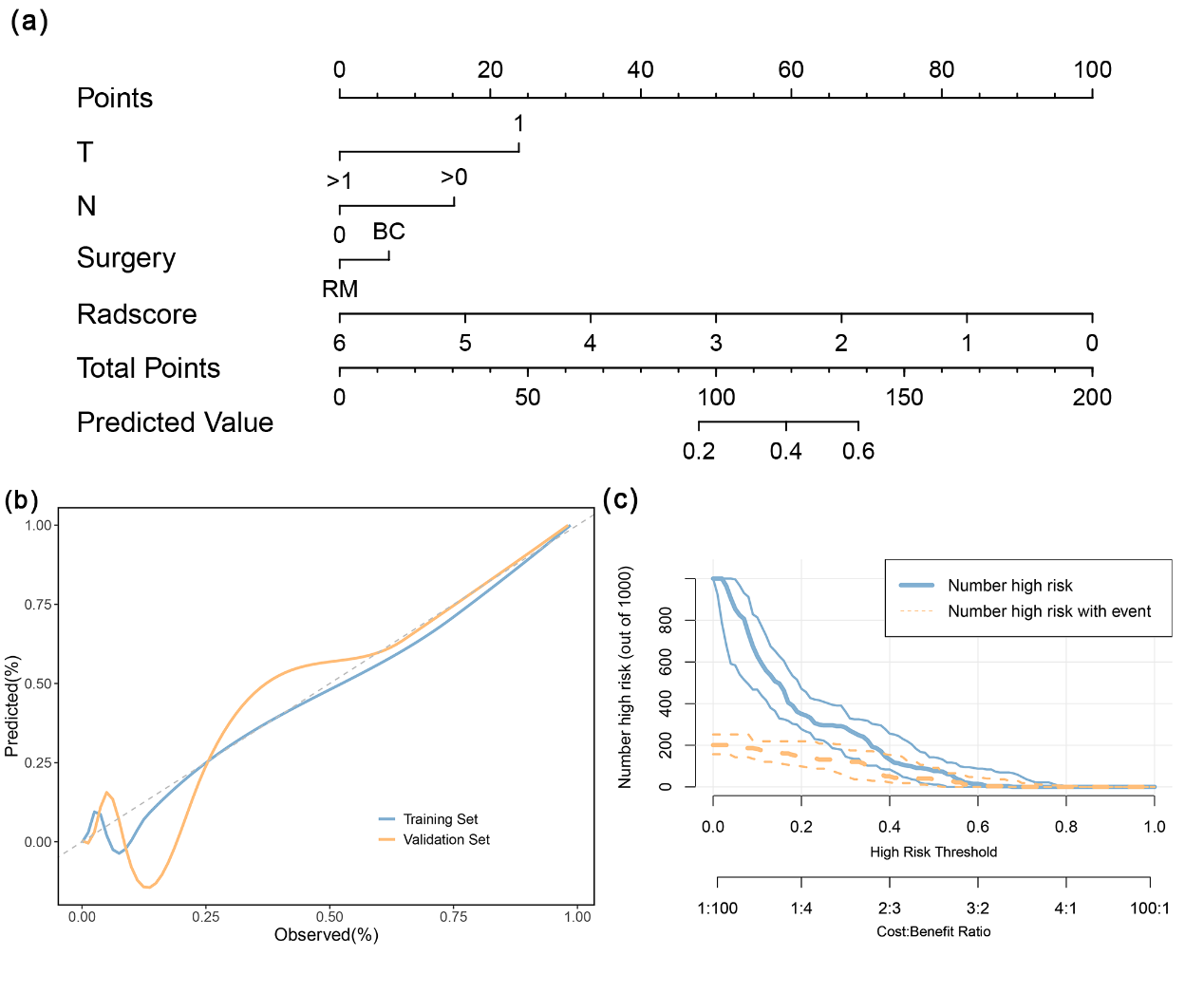


**Figure S2** **(a)** An individualized nomogram based on rad-score and clinical features for 5-fields plan. **(b)** Calibration curve of the nomogram. **(c)** Clinical impact curves of the nomogram.


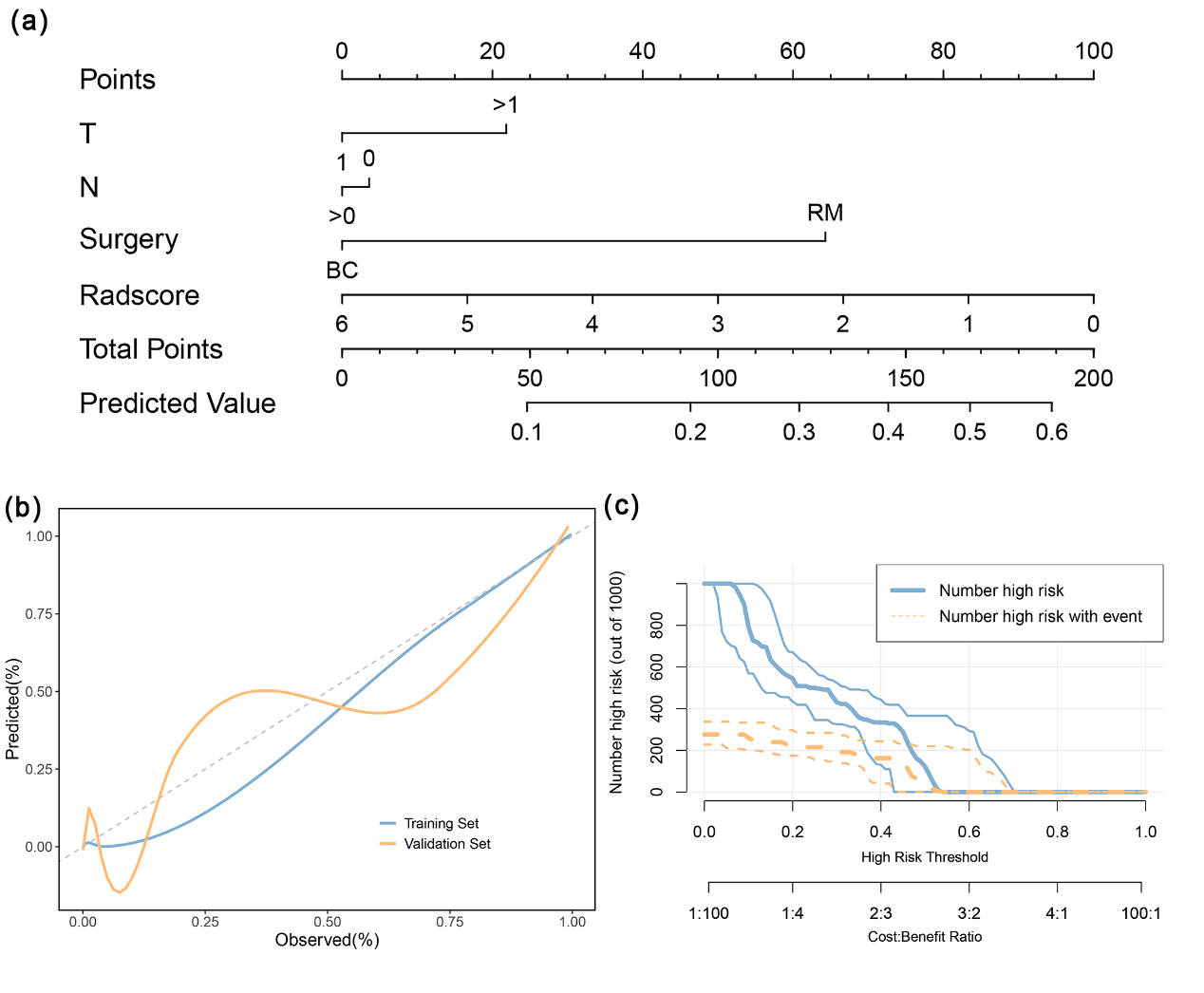


**Figure S3 (a)** An individualized nomogram based on rad-score and clinical features for 6-fields plan. **(b)** Calibration curve of the nomogram. **(c)** Clinical impact curves of the nomogram.


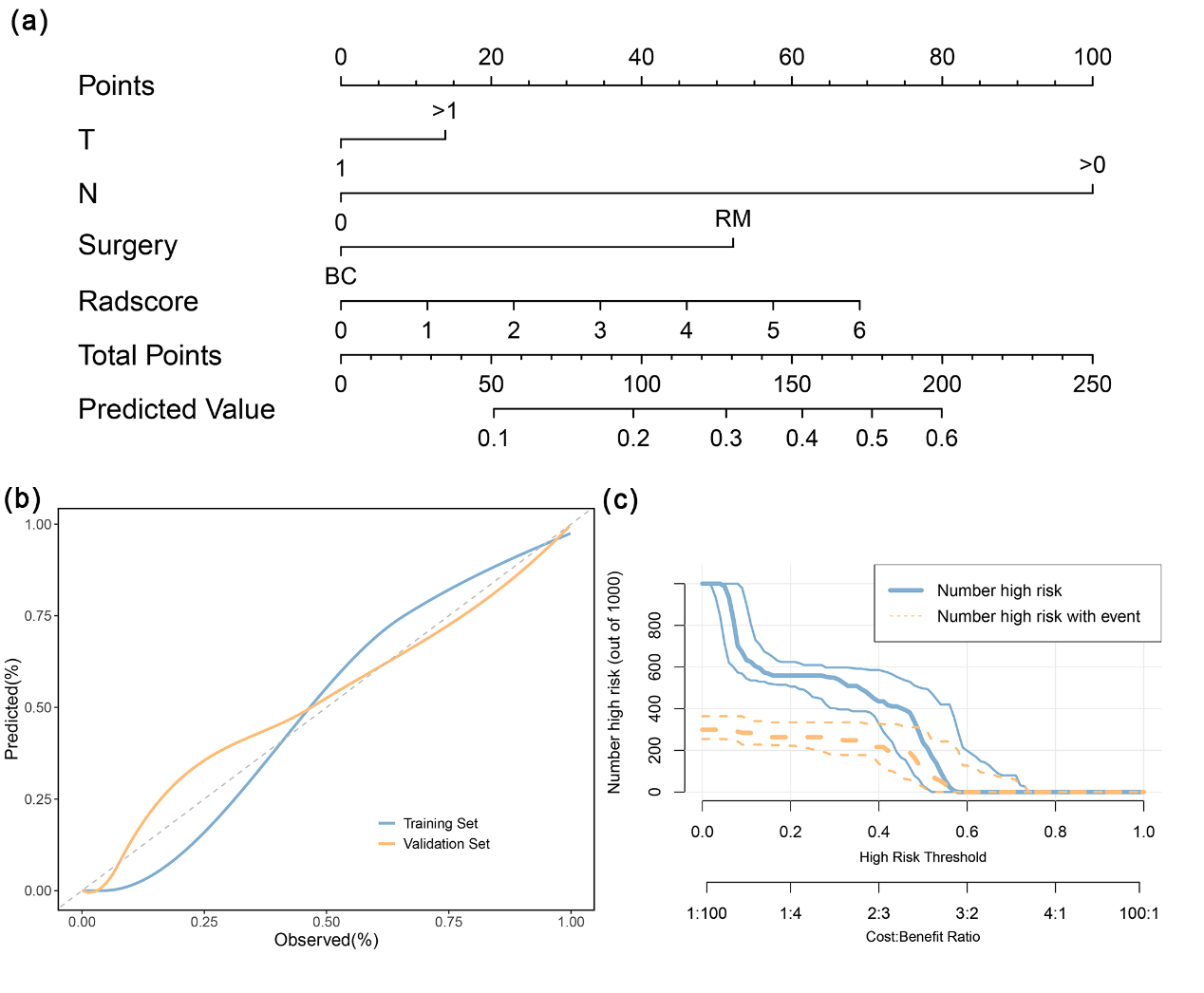


**Figure S4 (a)** An individualized nomogram based on rad-score and clinical features for 7-fields plan. **(b)** Calibration curve of the nomogram. **(c)** Clinical impact curves of the nomogram.
